# A cellular automaton model of crystalline cellulose hydrolysis by cellulases

**DOI:** 10.1186/1754-6834-4-39

**Published:** 2011-10-17

**Authors:** Andrew C Warden, Bryce A Little, Victoria S Haritos

**Affiliations:** 1 CSIRO Energy Transformed Flagship and CSIRO Ecosystems Sciences, PO Box 1700, Canberra, Australian Capital Territory 2601, Australia; 2 CSIRO Livestock Industries, FD McMaster Laboratory, Armidale, New South Wales 2350, Australia; 3 CSIRO Livestock Industries, Queensland Biosciences Precinct, 306 Carmody Road, St Lucia, Queensland 4067, Australia

## Abstract

**Background:**

Cellulose from plant biomass is an abundant, renewable material which could be a major feedstock for low emissions transport fuels such as cellulosic ethanol. Cellulase enzymes that break down cellulose into fermentable sugars are composed of different types - cellobiohydrolases I and II, endoglucanase and β-glucosidase - with separate functions. They form a complex interacting network between themselves, soluble hydrolysis product molecules, solution and solid phase substrates and inhibitors. There have been many models proposed for enzymatic saccharification however none have yet employed a cellular automaton approach, which allows important phenomena, such as enzyme crowding on the surface of solid substrates, denaturation and substrate inhibition, to be considered in the model.

**Results:**

The Cellulase 4D model was developed *de novo *taking into account the size and composition of the substrate and surface-acting enzymes were ascribed behaviors based on their movements, catalytic activities and rates, affinity for, and potential for crowding of, the cellulose surface, substrates and inhibitors, and denaturation rates. A basic case modeled on literature-derived parameters obtained from *Trichoderma reesei *cellulases resulted in cellulose hydrolysis curves that closely matched curves obtained from published experimental data. Scenarios were tested in the model, which included variation of enzyme loadings, adsorption strengths of surface acting enzymes and reaction periods, and the effect on saccharide production over time was assessed. The model simulations indicated an optimal enzyme loading of between 0.5 and 2 of the base case concentrations where a balance was obtained between enzyme crowding on the cellulose crystal, and that the affinities of enzymes for the cellulose surface had a large effect on cellulose hydrolysis. In addition, improvements to the cellobiohydrolase I activity period substantially improved overall glucose production.

**Conclusions:**

Cellulase 4D simulates the enzymatic hydrolysis of cellulose to glucose by surface and solution phase-acting enzymes and accounts for complex phenomena that have previously not been included in cellulose hydrolysis models. The model is intended as a tool for industry, researchers and educators alike to explore options for enzyme engineering and process development and to test hypotheses regarding cellulase mechanisms.

## Background

As an abundant and renewable material, biomass has been investigated extensively as a precursor of biofuels that can replace current fossil oil-based transport fuels and reduce greenhouse gas emissions. Biomass occurs in a variety of forms but commonly includes cellulose, a highly stable polymer of glucose, as a major component. The saccharification of cellulose, breaking the polymer into monosaccharides, yields glucose, which can undergo microbial fermentation to biofuels such as ethanol and butanol, or be used as a feedstock for the production of lipids and chemicals. Achieving efficient saccharification from complex biomass is challenging, and one of the main factors retarding the commercial biochemical method of cellulosic fuels and chemicals production [1].

Enzymatic saccharification of cellulose is achieved through the combined actions of 1,4-β-D-endoglucanases (EG) and 1,4-β-D-exoglucanases (or cellobiohydrolases - CBHI and CBHII), collectively known as cellulases. The products of these combined activities are the disaccharide, cellobiose, and polysaccharides such as cellotriose and cellotetraose, which are further reduced to monosaccharides by β-glucosidase (BG), and sometimes also by EG and CBH. The combined activities of these enzymes have been previously described in functionally-based cellulase models [2-5], and the various approaches taken have been recently reviewed [6]. Zhang and Lynd [7] have constructed the most detailed model to date which, for the first time, made extensive use of published experimental data as validation sets. While excellent progress towards a universally applicable cellulase model has been made with each of the abovementioned reports, none have addressed key phenomena such as crowding of substrate sites (competitive adsorption) during high enzyme loading, product and substrate inhibition, nonproductive adsorption and enzyme deactivation within a single model. This is likely due to the complexity encountered even when looking at a small part of the whole system and the prohibitive complexity of the differential equations required to fully describe these phenomena.

An approach that addresses this complexity is modeling using cellular automata (CA), that is, treating cellulose and enzymes as individual entities and assigning behaviors to each based on published kinetic values. CA methods are being applied to an ever-increasing variety of biological [8-11] and non-biological [12,13] systems to gain insights into the mechanisms responsible for emergent properties in complex systems. They differ from traditional differential equation-based modeling in that they provide facile representation of spatial relationships in two or three dimensions and allow a detailed examination of the effects of small (or large) changes in rules of thumb governing how automata move and interact. A valuable advantage of using CA models is that multiple hypotheses can be tested in the system, such as examining the possible outcomes from a reduction of the binding affinity of an enzyme to a solid substrate and the relieving of the effects of crowding at the substrate surface. Additionally, visual inspection of a three-dimensional model provides insights into possible mechanisms and physical interactions that may not have been realized in purely mathematical models, and also greatly aids troubleshooting during development.

Our aim was to develop a cellulose-cellulase model that provides control over many of the physical and chemical variables occurring during the enzymatic saccharification of cellulose in either the laboratory or on an industrial scale. In this report we demonstrate the model's utility in hypothesis-testing as a guide to cellulase enzyme research and improvement in rates of cellulose hydrolysis. The model is designed to be applicable across a broad range of cellulases, rather than tailored to describe enzymes from any particular source, and requires no previous knowledge of programming or scripting, or the use of specialist software packages.

## Results

### Base case

**Figure 1:**
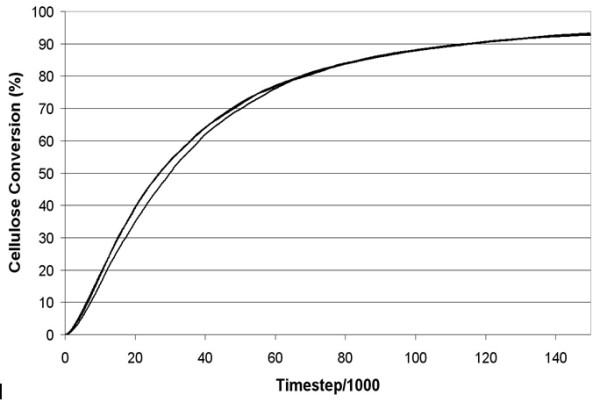
**Cellulose hydrolysis curves produced by replicated experiments using the base case parameters shown in Table 1 which closely match the hydrolysis curve shown in **[20].

The base case for the model utilizes parameters selected from the literature describing the cellulases of *Trichoderma reesei*. The resultant cellulose conversion curves produced by the model using the base case parameters closely mimic the shape of those found experimentally, being characterized by a fast initial rate of hydrolysis followed by a marked reduction in hydrolysis rate as cellulose degradation progresses [14] (Figure 1). One departure from the *T. reesei *system is our selection of a small enzyme 'footprint' on the cellulose surface, which has enabled simulations to be run in a reasonable timeframe by reducing the crowding effect.

#### Varying the enzyme concentrations of EG and CBH

**Figure 2:**
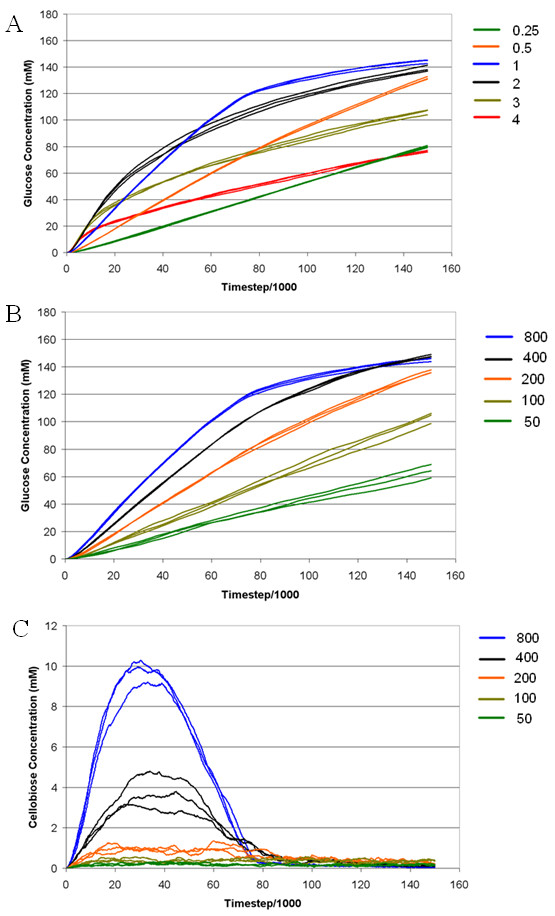
**The effect of enzyme concentration on the release of saccharification products from cellulose over time as determined by Cellulase 4D**. **(A) **Rate of glucose production over time from cellulose treated with various concentrations of cellulase mixture. The base case covering the concentrations of all enzymes is indicated by the number 1 and proportions of this amount given in the legend **(B) **Rate of glucose release at varying endoglucanase concentrations (between 800 and 50 mM) where the amounts of other cellulases are added as per the base case. **(C) **Cellobiose production rates under the same conditions described in (B). The legend showing different enzyme concentration (mM) settings used in the simulation is shown to the right of each graph and base case values are given in Table 1.

Reducing or increasing the concentration of the enzyme mixture relative to cellulose while maintaining the same concentration ratios of the enzymes generally had a negative effect on glucose release rates at the early stages of the simulations (Figure 2A). However, the glucose concentration at the end of the simulation period was not significantly different (*P *= 0.02) between the base case and doubling the loading of the enzyme mixture. The final glucose production at all other loading levels tested in the simulation (3×, 4×, 0.5× and 0.25×) were significantly reduced (*P *< 0.05) compared with the base case. In the follow-up trial, where the concentration of EG alone was reduced from the base case level of 800 to 400 mM while holding the other enzymes at base case levels, no significant effect was noted on final glucose concentrations (Figure 2B). However, further concentration reductions of EG to 200 mM and below did significantly reduce the final glucose yield (*P *< 0.05) and the rates of glucose release. Examination of the production of cellobiose with a reduction in EG concentration showed a negative effect on yield, as shown in Figure 2C.

#### Varying the adsorption strengths of EG and CBH

**Figure 3:**
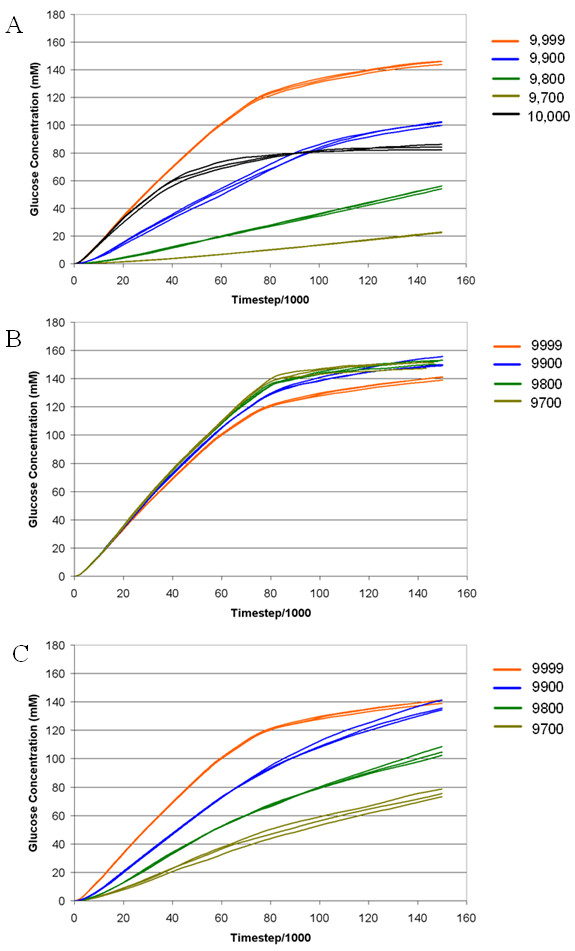
**The effect of adsorption strength of cellulases on glucose production from cellulose over time as modeled by Cellulase 4D; parameters were varied between 10,000 and 9,700 and a simulation conducted in triplicate for each parameter change**. **(A) **EG, CBHI and II varied by same parameter change **(B) **CBHI adsorption strength varied only **(C) **EG adsorption strength varied only. The legend showing different parameter settings is given to the right of each graph. CBH: cellobiohydrolase; EG: endoglucanase.

As determined by experiments conducted with Cellulase 4D, the adsorption strength of cellulases has a major influence on the rate of cellulose hydrolysis. The production of glucose was significantly reduced (*F *3618.74; *P *< 0.05) at the end of the simulation when adsorption strength was either increased or decreased compared to the base case (Figure 3A), although the pattern of glucose production was different for each. Where enzymes bound irreversibly to cellulose (adsorption strength = 10,000), hydrolysis was rapid until a limit was reached and thereafter the production of glucose plateaued (Figure 3A). A reduction in adsorption strength of all enzymes below that for the base case (that is, below 9,999) resulted in a significantly lower production of glucose and a slower rate of hydrolysis (Figure 3A). The reduction of the adsorption strength parameter for CBHI from 9900 to 9700 whilst holding the other cellulases at base case values caused small but significant increases (*P *< 0.001) in overall glucose amounts at the end of simulation (Figure 3B). By contrast, when the only the adsorption strength for EG was varied, there was a significant reduction (*P *< 0.02) in glucose production rate where absorption strength was set at 9800 and below (Figure 3C).

#### Varying the catalytic activities of EG and CBH

**Figure 4:**
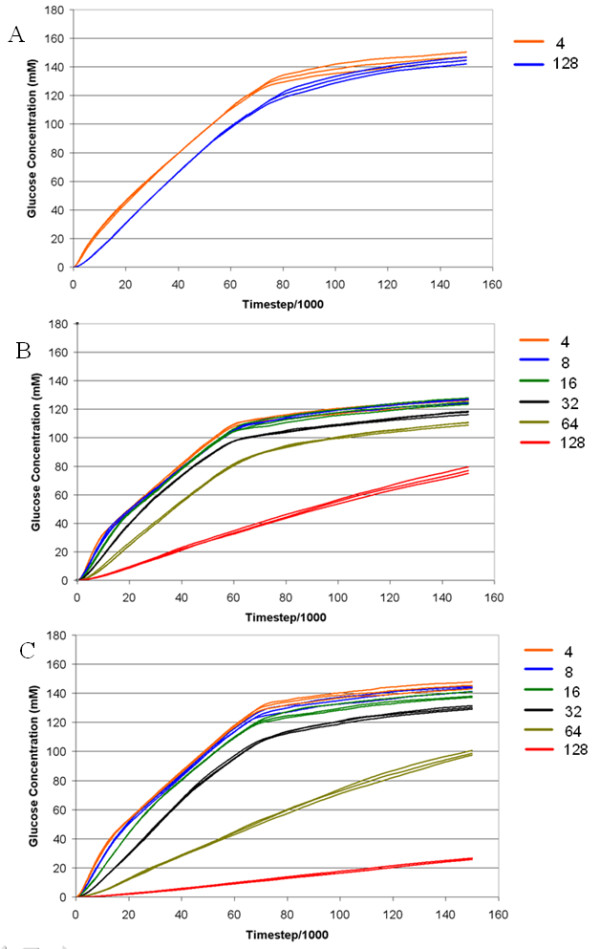
**The effect of altering the EG reaction period on the release of glucose from cellulose under conditions of different adsorption strength parameters and reaction periods of between 4 and 128 ms**. Absorption strengths of EG and CBHs are (**A**) 9999, **(B) **9900, and **(C) **9800. EG activity period is given in the legend to the right of each graph (ms). CBH: cellobiohydrolase; EG: endoglucanase.

In the first iteration of the base case, the reaction period for EG was varied between 1 ms and 128 ms at an adsorption strength of 9,999 to determine the effect on amount of glucose released of varying EG activity. No noticeable effect on the production of glucose was observed between these extremes of activity period (Figure 4A). The reaction period simulations were repeated at lower adsorption strengths for all cellulases as this has the effect of relieving any potential overcrowding of the cellulose surface, and a strong dependence of glucose production on EG reaction period emerged. At adsorption strengths of 9900 and 9800, a reduction in activity period from 100 ms (base case) to 64 ms and below caused significant increases (*P *< 0.05) in the amounts of glucose released (Figures 4B and 4C). Conversely, lengthening the activity period from 100 ms to 128 ms significantly reduced glucose production (*P *< 0.005). Thus, the slowing effect on glucose production rates of lower enzyme absorption strengths as shown in Figure 2A was counteracted by decreasing the EG reaction period.

**Figure 5:**
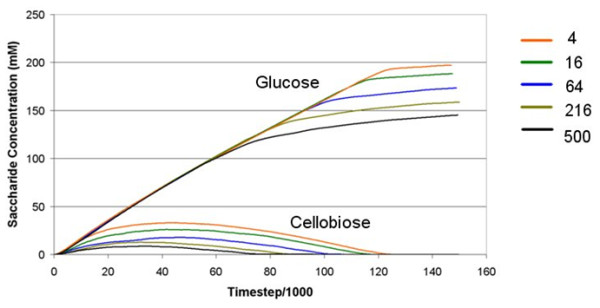
**The effect of decreasing the CBH reaction period on release of glucose and cellobiose from cellulose**. For clarity, a single representative experiment is shown for each reaction period in the figure. CBH activity period is given in the legend (ms) where 500 ms represents the base case value. CBH: cellobiohydrolase.

In the example where both CBHI and CBHII had their reaction periods reduced from 500 ms (base case) to 4 ms, a 125-fold improvement, a highly significant increase, in the rate of glucose and cellobiose production was observed (Figure 5). The cellobiose production rate dropped as hydrolysis progressed but, interestingly, there was no noticeable effect on the rate of glucose production until after this occurred. Decreasing the reaction period for CBH resulted in an enhanced final glucose yield at the end of the simulation period whilst maintaining the adsorption strength for all enzymes at base case value.

## Discussion

Cellulase 4D incorporates a novel approach to modeling the enzymatic hydrolysis of cellulose by cellulases, by introducing a cellular automaton method to describe the surface interactions combined with a mean field method to account for the solution phase kinetics. This has allowed an examination of the physical aspects of enzyme behavior, such as crowding and the non-productive occupation of reaction sites, that are often overlooked using more traditional cellulose hydrolysis modeling approaches. In Cellulase 4D, the simulated cellulose hydrolysis curves show a decrease in the rate of glucose production over time (Figure 1) primarily due to decreased cellulose surface area, increased enzyme crowding on the remaining surface and, to a lesser extent, product inhibition. The curve shapes produced by the model are typical of those produced using experimental data (for examples, see [14,15]) albeit over shorter time periods (as discussed above). This may point to a problem with most current models in that they do not take into account such physical characteristics of the system and may potentially lead to 'overfitting' of data to accommodate equations designed to describe solution phase behaviors.

The three scenarios modeled in Cellulase 4D examined the consequences for cellulose hydrolysis of the complex interplay between the affinity of an enzyme for the cellulose surface, the catalytic activity of each enzyme, the concentration of enzyme present and the interactions of enzymes with each other on the surface in the form of crowding. The scenario modeling results for cellulose hydrolysis rates with different enzyme loadings indicate a potential optimum enzyme loading of between 0.5 and 2 of the base case concentrations. At this loading, a balance was obtained between crowding on the cellulose crystal, whose accessible surface area was being gradually reduced, and having insufficient enzyme to adequately utilize the available surface area. Reducing the EG concentration relative to the other enzymes had an unexpectedly negative effect on the cellobiose production over time, suggesting that much of the cellobiose production is actually dependent on EG activity. This can be partially explained by the behaviors ascribed to EG, where the enzyme locates a reaction site, acts and then resumes a random walk in close proximity to another appropriate reaction site on the same chain, which may be as close as two glucose molecules away. An additional reason for the reduction in cellobiose production could be the concomitant reduction in the well-documented synergy between the EG and CBH components [16], with fewer reducing and non-reducing ends being produced, hence less potential reaction site from which the CBHs can commence their processive action. Similar hydrolysis product distributions have been produced by EGs in experimental studies utilizing carboxymethyl cellulose, although the proposed reason was the ability of one of the EGs to hydrolyze cellopentaose and cellotetraose [17].

Modifying adsorption strengths of all surface acting enzymes resulted in large effects on the cellulose hydrolysis rate (Figure 3). Irreversible sorption rapidly caused enzyme crowding and no further hydrolysis occurred, whereas a reduction in absorption strength caused a dramatic reduction in hydrolysis rates. However, when the adsorption strengths of CBHI and EG were modified individually, the effect on cellulose hydrolysis was much lower than when all the enzymes' adsorption strengths were modified simultaneously. This was most likely a simple reflection of fewer enzymes being varied, therefore having a smaller overall influence on changes in crowding effects. Adsorption strength relates to crowding of the cellulose surface, therefore adjustment of the adsorption strength of some cellulase enzymes through modification of the carbohydrate binding module (CBM) may present a target for enzyme engineering in some cellulase systems.

The slow catalytic rates of CBH enzymes (*k*_cat _is approximately 2 to 0.1 s^-1^) [18] and their role as the rate-limiting agents of cellulose hydrolysis have been discussed, however, less is known about the potential effects on the overall cellulose degradation rate of varying EG catalytic activity. Hence, we designed experiments to assess the effectiveness of increasing catalytic activities of both groups of enzymes. EG reaction time was found to heavily influence the overall rate of glucose production, but only while a moderate adsorption strength for the enzyme was employed. There was a distinct and unavoidable trade-off between the time taken for EG enzymes to interact with the surface long enough for sufficient reaction points to be located, and the resulting overcrowding that hampered access to those points. Where adsorption strength for the enzymes was strong, all appropriate reaction sites became irreversibly occupied by enzymes that were not able to act upon those points.

It has been previously reported that enzyme synergism decreases at higher enzyme loadings, and that at saturation loading, cellulose hydrolysis becomes inhibited [19]. Earlier studies looking at the effect of enzyme loading on the rate of hydrolysis have shown a leveling off in hydrolysis rate with increasing enzyme loading [20], however the loading ranges tested are generally not as broad as those tested in our scenarios. In the model, we see a significant reduction in the hydrolysis rate when we depart from the 'ideal' enzyme loading (Figure 2). The dependence of hydrolysis rate on enzyme loading is attributable to surface saturation being achieved at high adsorption strengths, making crowding the rate determining parameter, whereas at lower adsorption strengths, the actual reaction rate of the EG enzymes was the main contributor. A likely consequence of strong adsorption of enzymes is that CBHs became hemmed in on accessible chains where EGs would otherwise be able to act, and EGs became crowded onto reducing and non-reducing ends where CBHs would otherwise be able to act, markedly slowing cellulose degradation. This saturation point occurs slightly later at the lower adsorption strength and there is more cellulose hydrolyzed at this point. When adsorption strength was reduced, slower acting EG slowed the overall rate of glucose production significantly, with only one-third of the glucose being produced at the end of simulation at an adsorption strength of 9800 compared to that at 9900.

However, improvement to the CBHI activity period had a major effect on the overall glucose production rate and resulted in the greatest increase among the scenarios tested. A reduction in CBH reaction rate had no effect on the rate of glucose production, due to enzyme crowding, until after the rate of cellobiose production had slowed. At this point, BG activity was no longer the rate-limiting step in glucose production. During the initial stages of hydrolysis, the CBHs released more cellobiose to solution before crowding became too severe and slowed reactions, but this resulted in higher levels of glucose produced by the end of the simulation as BG subsequently hydrolyzed the accumulated cellobiose.

EG had a strong influence on the overall rate of hydrolysis in our model. Although this may be partially the result of the much slower acting CBH enzymes, it is also possible that the longer polysaccharides released from cellulose by the EG do not dissociate from the surface as readily in the physical system compared to the model. The continued association of 4-, 5- and 6-sugar polysaccharides with the cellulose surface would likely have the effect of hindering access of EGs to appropriate surface reaction sites, and also reduce the apparent rate of glucose production by being less accessible to enzymes that further degrade them to shorter polysaccharides and, eventually, glucose. Additionally, any saccharides that are inhibitors of the surface adsorbed enzymes would have a much higher apparent concentration from the perspective of surface adsorbed EG and CBHs compared to those same inhibitors in solution.

Owing to the high computational cost of such a large CA model and the need for pragmatic simulation times, certain parameters were exaggerated or undervalued in the model. The smallest plausible enzyme spatial requirement was utilized, which greatly facilitated shorter simulations; it was assumed that no enzymes were denatured on the cellulose surface and high adsorption probabilities (90%) were used. The small spatial requirement allowed EG to cleave points in the cellulose chain quite close to each other, which resulted in the rapid formation of the short, soluble polysaccharides. The time taken for each simulation could potentially be shortened by modifying the algorithms for parallel processing which would greatly reduce the time required.

The model presented herein is a framework upon which further refinements can be made and currently represents only the fundamental aspects of cellulase activity. Recently, another class of cellulases has come to light that employ reactive oxygen species to attack glycosidic bonds in cellulose [21,22]. Future versions of the model will include the capability to model the action of such enzymes, particularly as more information comes to light about their kinetics and modes of action. The addition of lignin and hemicellulose components to the three-dimensional model would be useful for applicability to natural substrates although it would be vastly more complicated. Additionally, the model could be coupled in a modular manner to large scale process models. Control of parameters such as temperature and viscosity, which influence enzyme mobility, the rate of enzyme deactivation and adsorption equilibria would aid process design. There is also the potential, in the future, to implement a more detailed and flexible inhibition model.

## Conclusions

We have described the cellular automaton model Cellulase 4D that simulates enzymatic hydrolysis of cellulose to glucose by surface and solution phase-acting enzymes. After establishing a base case for cellulose hydrolysis by implementing kinetic values obtained from the literature, we examined the effect on rates of degradation and saccharide production of altering key enzyme parameters. Adsorption strength of EG, reaction periods for CBH and enzyme loadings used were identified as having significant effects on cellulose hydrolysis.

## Methods

### Model overview

Cellulase 4D describes the decomposition of a crystal of cellulose by the actions of a mixture of enzymes that work in concert on both insoluble and dissolved substrates, eventually yielding glucose and some residual polymeric material. Many parameters governing the behaviors of the enzymes can be modified to allow the input of new enzyme properties, or for hypothesized activities to be tested. A 'single-enzyme' approach was suggested by Gentry and co-workers [23,24] where they proposed a 'microscopic model' of enzyme kinetics in solution that provided a more flexible and robust framework within which to examine non-ideal systems. In our model, default values are provided for each parameter. These were primarily derived from experimentally-determined literature values and based on the catalytic mechanisms and activities of the most widely studied cellulases - those derived from *T. reesei *(*Hypocrea jecorina*). The main outputs of the model are the concentrations of saccharide species in solution over time and the percentage of the initial cellulose that is hydrolyzed in a given time period.

In Cellulase 4D, a single virtual cellulose crystal and enzymes exist in a three-dimensional space with periodic boundary conditions, that is, an enzyme exiting one side of the space will reappear on the opposite side of the space. This rule maintains a constant concentration of enzyme and is an effective way to simulate a representative part of a larger system. The model incorporates the activities of both the adsorbed enzymes (EG, CBHI and CBHII) and those working in solution (EG, CBHI, CBHII and BG). Each enzyme and its interactions with the range of substrates and inhibitors produced are considered separately and their characteristics are described in the relevant sections. For many of the model parameters pertaining to the enzymes, such as product inhibition constants or the time taken to complete a reaction on the cellulose surface, the user can choose to either input default settings based on experimentally-determined values or they can input new hypothetical values to model the effect of those changes on cellulose hydrolysis rate and the levels of the various species in solution. Other parameters, such as the size of the enzymes' 'footprint' and the size of the three-dimensional space surrounding the cellulose, are fixed and unable to be altered. Outputs, such as saccharide species in solution and the percentages of cellulose hydrolyzed and of enzyme amount adsorbed to the surface, are plotted graphically and also saved in tabulated form as the simulation progresses.

Factors that have been accounted for in the model at the level of an individual enzyme include specific activities and substrate preferences of each of the component cellulases; enzyme promiscuity, as some cellulases can hydrolyze both soluble and insoluble substrates; inhibition of enzyme activity, both in solution and on the cellulose surface, by reaction products; strength of adsorption and probability of enzymes adsorbing to the cellulose surface once in proximity; and the potential for crowding at higher concentrations of enzyme.

Prior to initiating simulation, kinetic parameters, such as *K*_m_, *k*_cat _and K_i _of all enzymes with respect to activities, if any, on soluble saccharides, are selected, and the kinetic parameters of CBHI, CBHII and EG with respect to the cellulose are established. The option for all enzymes to be either inhibited by, or active upon, any or all soluble polysaccharides is available if the user wishes to incorporate solution activity into the model. Enzymes also have a 'lifetime' (see Denaturing below) after which they become denatured but either remain in solution or adsorb to the surface and contribute to crowding. Once adsorbed to the surface, the enzymes do not diffuse into solution unless they are adsorbed to a polysaccharide that is solubilized through the action of another enzyme.

### Cellulose

The virtual cellulose crystal used in the model is based on a highly structured cellulose crystal, such as that produced by *Valonia macrophysa *and imaged by Lehtiö *et al*. [25] in a report demonstrating the preference of CBMs for particular faces of the crystals, although, in this model, we have not given enzymes preference for particular faces. *Valonia *cellulose is a very highly structured form of cellulose, with each single crystalline microfibril having an approximate cross-sectional area of 20 × 20 nm [26] and containing around 1200 to 1400 individual cellulose chains [27]. Higher plant cellulose microfibril diameters are typically smaller than *Valonia; *as small as 3 nm and containing around 30 to 36 cellulose chains [28].

**Figure 6:**
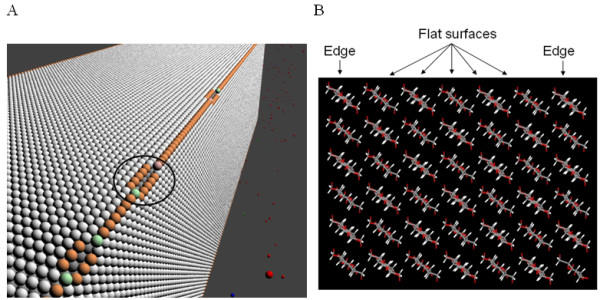
**The cellulose crystal which is the substrate for enzymatic attack in Cellulase 4D**. **(A) **Three-dimensional representation of a cellulose rod where individual glucose molecules are represented by colored spheres. Orange spheres are the accessible edge glucose moieties in the cellulose chain; pink and green spheres signify the reducing and non-reducing ends, respectively, of the cellulose chain. The white spheres are inaccessible subsurface, flatsurface residues. The circled section indicates the site of endoglucanase hydrolysis of the cellulose chain at two positions five glucose units apart, leaving a reducing and non-reducing end to the chain. The released cellopentaose was solubilized and not indicated in the figure. **(B) **A view down the length of a smaller fragment of cellulose generated from its crystal structure, showing chains that are more exposed and hence more accessible to cellulases than those that are bordered by four adjacent chains.

In the model, the virtual cellulose consists of glucose chains running the full length of the crystal that are characterized as either 'edges', 'flatsurfaces' or 'subsurfaces' (Figure 6A and 6B). We deemed that the cellulose chains on the edges are the most accessible to EG and CBHs as they are not flanked by cellulose chains on both sides and are less well bound to the cellulose. Hence, we have determined these to be the only chains available to EG and CBHs in the model. Once a CBH hydrolyzes a portion of a cellulose chain, it exposes two more chains as edges that can then be accessed by the hydrolytic enzymes as they are no longer flanked on all sides (Figure 6A). This is an alternative mechanism for exposing additional accessible cellulose surface to that discussed recently by Arantes and Saddler [14], where it was suggested that the CBM buries itself in the cellulose at hydrolyzed or amorphous sections, allowing water to penetrate the bulk, hence providing more accessible cellulose chains. The actual nature of the mechanism whereby additional cellulose chains are made available is largely unknown, in spite of significant investigations.

As hydrolysis of glucose residues from cellulose progresses through the activity of EG and CBHs, shorter saccharides appear on the cellulose surface. Where the chain length is six glucoses or less, the saccharide is considered soluble in the model and contributes to the overall sugar species in solution, where it can then be further hydrolyzed by enzymes in solution. Any enzymes that were located on the dissolving saccharide are considered to be desorbed from the cellulose.

### Progression, adsorption and reaction time behaviors for enzymes

Enzymes diffuse through solution according to a 'random walk' algorithm [29] and adsorb to the cellulose according to an 'adsorption probability parameter' and whether the prospective adsorption site is occupied by another enzyme or not.

#### Random walk

A random walk is used to mimic Brownian motion of enzymes in solution. At each time point, a random vector in three-dimensional space is chosen and the enzyme moves 5 Å in that direction. While it is understood that an enzyme will diffuse much further than 5 Å in 1 ms in aqueous solution, we have found our approximation gives a satisfactory representation of previously reported [30] adsorption profiles for cellulase enzymes when used with an appropriate adsorption probability parameter. It has been shown that enzymes generally take several minutes for equilibrium to be reached, so for real systems, it takes a great number of collisions before a successful adsorption event. We found that allowing an enzyme to adsorb every time it came within a certain distance of the cellulose surface gave unrealistically rapid adsorption rates. The implementation of an adsorption probability parameter allows an adsorption rate to be chosen [30] that more closely mimics the real system. All enzymes that act upon the cellulose are deemed to move about the surface in a random walk [31,32] in steps equating to the dimensions of one glucose molecule (5 Å) per time-step. It is noteworthy that Beldman *et al. *[33] found no relationship between enzyme mobility on the surface and the tightness/irreversibility of binding, and the behavior of the enzymes in our model reflects this. As reported by Jervis *et al*. [34], the diffusion rates of *Cellulomonas fimi *cellulases on the cellulose surface ranged from 2 × 10^-11 ^to 1.2 × 10^-10 ^cm^2^/s which equates to around 200 Å^2 ^(10 square glucose molecules) per millisecond. Our selection of 5 Å increments are supported by molecular dynamics simulations reported by Bu *et al*. [35] that indicated that the carbohydrate binding domain has potential energy minima every 5 and 10 Å along the cellulose surface, corresponding to the lengths of glucose and cellobiose, respectively.

#### Activity period

Once a reaction site is located on the cellulose rod and the enzyme becomes active, the time taken for the reaction to go to completion is calculated by selecting a random point under a normal distribution curve centered on 1/*k*_cat _for the enzyme in question, an activity period. Both the *k*_cat _and standard deviation for each enzymatic reaction occurring in solution are selected before the simulation is initiated.

#### Adsorption strength

Cellulases display many different adsorption characteristics [33,36-38] ranging from loose, reversible adsorption to irreversible adsorption. In some cases, cellulase adsorption to cellulose has also been shown to be influenced heavily by glucose and cellobiose in solution, with no binding to surfaces seen at cellobiose concentrations above 150 g/L [39]. While we have not implemented a concentration-dependent inhibition of adsorption by product molecules, to account for this broad range of behaviors, we implemented an adsorption strength parameter between 1 and 10,000 that represents the probability that an enzyme will desorb from the surface at any particular point in time. For instance, an adsorption strength of 10,000 indicates irreversible binding. Conversely, an adsorption strength of 1 indicates essentially no binding. An adsorption strength of 5,000 results in the enzyme (provided it is not currently participating in a reaction) having a 50% chance of desorbing from the surface at any particular time-step, which is still quite weak binding.

#### Adsorption probability

This is the probability of adsorption of an enzyme in solution that comes into close proximity of the cellulose surface, and is dictated by a parameter chosen for each enzyme prior to running a simulation. It is not expected that every collision of an enzyme with the cellulose would result in a successful binding event. This also provides the ability to slow down the overall rate of adsorption if users are modeling systems with slower adsorption profiles. When a desorption event occurs, determined by the adsorption strength parameter (see above), the desorbed enzyme is returned to the solution, increasing the concentration of that enzyme in solution. This sets up a dynamic equilibrium between adsorbed enzymes and those in solution.

### Endoglucanases

EGs act by hydrolyzing cellulose chains at random positions, creating reducing and non-reducing ends at the cleavage point. The behavior of EG in the model is described as follows. Once the EG locates an appropriate reaction position on cellulose and has become active (determined by a 'reaction probability' variable - see Equation 1), it remains stationary until the activity period has elapsed. It then performs the hydrolysis, its state is then reset to 'inactive', and it resumes a random walk pattern on the cellulose surface. EG activities are diverse, also acting on soluble substrates in solution such as cellotriose, cellotetraose, cellopentaose and cellohexaose to smaller polysaccharides [40-42] and also cellobiose to glucose [43]. The k_cat _values for the hydrolysis of cellobiose, cellotriose, cellotetraose, cellopentaose and cellohexaose are available as default settings or user-selected for EG in the model prior to each simulation. In the cases of cellotetraose and longer polysaccharides, the hydrolysis point along the chain is chosen at random in the model. For instance, a cellopentaose can be hydrolyzed to cellobiose and cellotriose species, or glucose and cellotetraose species with equal probability.

### Cellobiohydrolase

CBHI and CBHII are processive cellulases that hydrolyze cellobiose units from a cellulose chain, starting from either a reducing end (CBHI) or a non-reducing end (CBHII) that occurs where the cellulose chain is cleaved. After CBHs adsorb to the cellulose surface, they move about it in a random walk in increments of 1 glucose (5 Å) until a break containing a valid reaction site for the enzyme is located. Kipper *et al. *[18] examined the kinetics of cellobiohydrolase Cel7A using fluorescent model cellulose substrates. 'Processivity values', the average number of cellobioses cleaved from a cellulose chain in a single event by the enzyme, were reported for a range of substrates along with k_cat _values for the enzyme. For crystalline bacterial cellulose with a degree of polymerization of 1700 glucose units, the processivity value for Cel7A was 176 ± 20 with a k_cat _of 9.5 × 10^-2^/s. CBHs are slow catalysts in comparison with other enzymes; one cellobiose product is released from cellulose at intervals of between approximately 1 and 10 seconds [18] compared with k_cat _values in the order of 1 per 0.01 to 1 second for β-glucosidases [44].

In addition to random walk and adsorbing to the cellulose surface, CBHs (inactive) also have probabilities of desorbing from the surface or denaturing after a period of time. These are governed by the adsorption strength and 'lifetime parameters', respectively. CBH activity may also be inhibited by any or all of the saccharides in solution (see section Surface adsorbed enzyme inhibition), however, this does not affect their random walk, nor change their desorbing or denaturing probabilities in the model. If a CBH encounters another enzyme on the surface during the random walk phase it is precluded from moving to that point and will wait another program cycle (1 ms) before searching for a vacant adjacent point to move to. Likewise, if the active CBH encounters another enzyme during processive hydrolysis it is programmed to stop at that point until the cellulose chain becomes free of enzymes for a minimum distance of two glucose units ahead.

### Active and inactive enzymes

Enzymes on the surface of the cellulose are either inactive (in other words, not currently involved in a reaction) or active (complexed to the substrate and involved in a reaction). The length of time that a surface-adsorbed enzyme remains active after initiation of a reaction is user-determined prior to the start of the simulation via the activity period variable (described above), which is either set by the user or default values can be used. While active, surface adsorbed enzymes are stationary and while inactive, they are mobile.

### Denaturing of enzymes

Cellulases have been shown to denature during the course of cellulose hydrolysis [45]. Denatured enzymes are also known to inhibit binding of other cellulases to the surface and also to inhibit their activity once on the cellulose surface [30,46]. Each of the four enzymes involved in cellulose hydrolysis (EG, CBHI, CBHII and BG) is given a user-determined 'lifetime' (or a default value can be selected) prior to simulation which governs the average timespan for which they will remain active. Once this time period elapses, an EG or CBH enzyme in solution is programmed to continue a random walk until it encounters cellulose, then it will adsorb to the surface and remain stationary, preventing other enzymes from binding to, or acting at, that point. When an enzyme becomes deactivated while on the surface, it is programmed to remain stationary at that point and only desorb when the cellulose chain it is located on is hydrolyzed into a chain of six sugars or less by an EG or CBH and becomes soluble.

### Crowding of enzymes on the cellulose surface

Surface crowding is particularly difficult to account for using traditional Michaelis-Menten kinetics alone and most models thus far have chosen not to address this phenomenon explicitly. A coincident sharp decrease in the rate of cellulose hydrolysis in some cases with high enzyme loadings has previously been attributed to crowding [47,48] and one of the main aims of our work was to create a model in which this aspect could be easily explored.

Crowding (or jamming) of cellulases on the cellulose surface is a phenomenon that has received recent attention [49,50] and has been put forward as one of the major factors contributing to the well-known marked decrease in the rate of cellulose hydrolysis after a reasonable degree of conversion. Beldman *et al*. [33] showed that there is a high degree of variability between cellulase enzymes from *T. viride *regarding their adsorption profiles, that is, what quantity of enzyme will fit on a given amount of cellulose, with values ranging from 0.007 mg to 0.126 mg enzyme per mg Avicel. In order to estimate surface coverage by enzyme, we conducted a surface area analysis of Avicel using nitrogen adsorption and determined the surface area to be 3.74 × 10^-3 ^m^2^/mg. A discussion of the various merits and drawbacks of different methods for measuring cellulose surface area is beyond the scope of this report, and these aspects have been covered in detail elsewhere [51,52]. If an enzyme has a footprint of approximately 1500 Å^2 ^(calculated), then 1 mg of a *T. reesei *Cel7A catalytic domain (without CBM or linker) of an approximate molecular weight of 46,000 g/mol will have a footprint of 0.196 m^2^. So, for the lowest loading case in Beldman *et al*. [33] (0.007 mg enzyme per mg Avicel), there would have been 1.37 × 10^-3 ^m^2 ^footprint on a total surface of 3.74 × 10^-3 ^m^2^, or approximately one-third coverage. However, for the highest loading case of enzyme there would have been approximately six times coverage. In reality, this is likely to be significantly higher given the nitrogen probe provides an overestimate of the enzyme-accessible surface area owing to its small size compared to that of an enzyme. This could imply that EGs and CBHs can adsorb to cellulose in multiple layers [53,54] and still maintain their activities or, alternatively, that the CBMs are the only part of the enzyme close to the surface, with the catalytic domain floating a reasonable way from the surface tethered by the linker to the CBM. A very recent report by Igarashi and coworkers [55] has shed more light on different binding modes of Cel7A and we hope to incorporate these binding behaviors in further development of our model. A typical *T. reesei *CBM footprint is 260 Å^2^, approximately one-sixth that of the catalytic domain, which equates almost perfectly to total coverage of cellulose by one layer of CBMs at the highest loading described by Beldman *et al*. [33]. Thus, for the purposes of the model we have assumed that only one layer of enzyme can adsorb and that it is only the CBMs and not the catalytic domains, that can contribute to crowding on the surface. Therefore, we have implemented an enzyme spatial requirement equating to a 3 × 3 square of glucose residues. While this is slightly smaller than some CBMs and much smaller than catalytic domains, it allows the examination of enzyme crowding without exaggerating the effect in our model. Additionally, it reduces the time taken for a model run to achieve a reasonable degree of cellulose hydrolysis. Simulations run with larger spatial requirements, for example 4 × 4 and 5 × 5 glucose residues, took significantly longer owing to the greatly reduced rate of hydrolysis.

### Surface adsorbed enzyme inhibition

There are several different types of enzyme inhibition that have been experimentally observed for cellulase enzymes [56]. In the model we have restricted our consideration to competitive inhibition only, excluding non-competitive, uncompetitive and mixed inhibition mechanisms. The K_i _for a particular enzyme/inhibitor system in the model represents the inhibitor concentration at which the maximum rate of turnover (*k*_cat_) for the enzyme is reduced by half.

It is difficult to decouple the contributing effects that result in an experimentally determined K_i _value, that is, an inhibitor may be highly effective because it has a high probability of a successful binding event per collision, or it may have a lower probability of a successful binding event but it may have a much longer residence time in the enzyme, making it highly effective. Therefore, in the model we have simplified the potential inhibition of cellulose-adsorbed enzymes by implementing an activity probability variable (non-user chosen, between 0 and 1, see Equation 1) which changes according to the concentrations of the various inhibitors in solution. If there are no inhibitors present in solution, activity probability is set to 1. Equation 1 demonstrates how the activity probability for adsorbed, inactive EG is calculated at each timestep:

(1) EGAP=Ki(glu cose)[gluc]+Ki(glu cose)×Ki(cellobiose)[cellobiose]+Ki(cellobiose)×Ki(cellotriose)[cellotriose]+Ki(cellotriose)×Ki(cellotetraose)[cellotetraose]+Ki(cellotetraose)×Ki(cellopentaose)[cellopentaose]+Ki(cellopentaose)×Ki(cellohexaose)[cellohexaose]+Ki(cellohexaose)

**Figure 7:**
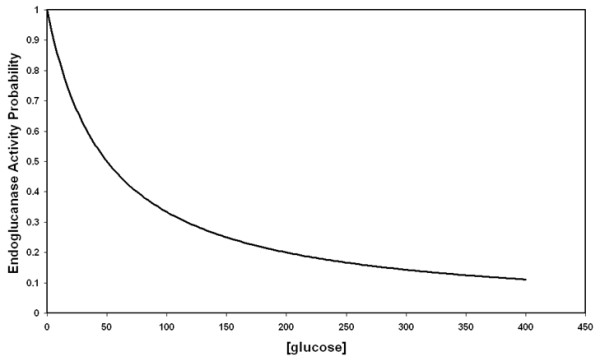
**An example of the variation of the activity probability variable for endoglucanase (EndoAP) with glucose concentration (K_i _= 50 mM) with the assumption that no other enzyme is inhibited**.

A graphical representation of how activity probability changes with glucose inhibitor concentration is shown in Figure 7.

### Solution phase reactions

Different reaction and inhibition behaviors were programmed for the soluble enzymes to those operating on the cellulose surface; they are not considered as individual enzymes but instead mean-field solution reactions whose parameters are chosen at setup. From these, a reaction velocity (*v*, in M.reactions per second) is calculated, which is then used to determine how many reactions will occur at each timestep. An example of how the solution reaction velocity between an endoglucanase and cellotriose is calculated, taking into consideration the concentrations of all other substrates and inhibitors, is shown in Equation 2:

(2) $$\begin{array}{l} v_{\left(EG-cellotriose\right)}=[EG_{inactive}]\times \frac{\left([cellotriose]\times k_{cat}\right)}{1000}\times ([cellotriose]+K_{m}\times \\ (1+(\frac{\left[cellobiose\right]}{K_{i(EG-glu\cos e)}})+(\frac{\left[cellobiose\right]}{K_{i(EG-cellobiose)}})+(\frac{\left[cellotetraose\right]}{K_{i(EG-cellotetraose)}})+\\ \left(\frac{\left[cellopentaose\right]}{K_{i(EG-cellopentaose)}})+(\frac{\left[cellohexaose\right]}{K_{i(EG-cellohexaose)}})\right) \end{array}$$

where count_EG _is the total number of EG in solution, k_cat _is the turnovers per second and *K*_m _and K_i _(both with units of mM) have the same definitions as understood in traditional Michaelis-Menten kinetics. The reaction velocity *v *is converted from M.reactions per second to reactions per millisecond (that is, per timestep) in the total volume and the appropriate polysaccharide concentrations are updated accordingly.

### Model outputs

**Figure 8:**
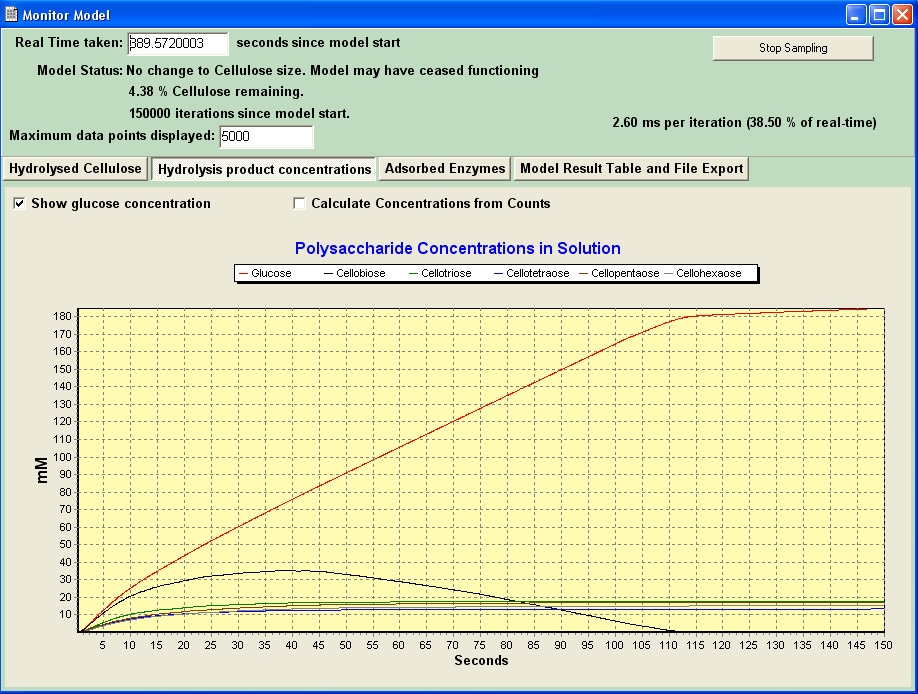
**Example output from Cellulase 4D showing real time graphical representation of the production of various saccharide species against time for an experiment**.

Outputs such as types and concentrations of saccharide species in solution against time, the percentage of initial cellulose hydrolyzed and enzyme amounts adsorbed to the surface are plotted graphically by the program and saved in tabulated form as the simulation progresses (Figure 8). Tabular data can also be exported in comma-separated-values (.csv) file format. Data is generated in real-time as the simulation progresses.

### Computer program flow

The following variables may be user-defined prior to simulation or default values selected:

• Cellulose dimensions (x, y and z in 5 Å increments)

• All enzyme concentrations (mM)

•* k*_cat _and *k*_catSD_: Average number of reactions per second per enzyme and the standard deviation. All enzymes have the option to be active on all substrates, with the exception of BG on cellulose.

• K_i_: All enzymes may optionally be inhibited by all saccharide species up to celloahexaose.

• Adsorption strength (from 0 to 10,000 with 10,000 being irreversible binding) for EG, CBHI and CBHII only

• Adsorption probability: The probability that an enzyme will bind to the cellulose surface when within 20 Å.

• Lifetime: All enzymes (seconds)

### Scenarios

Three scenarios were investigated which examined sensitivities in the model; these involved varying enzyme concentrations, adsorption strengths of EG and CBH and catalytic activities of EG and CBHI, and monitoring the rate of release of saccharides over time. The experimental designs are detailed below and each test of conditions was examined in triplicate simulation experiments. Firstly, a base scenario that employed average conditions and kinetic parameters from *T. reesei *cellulases [18,34,57-60] was established with the parameters given in Table 1.

**Table 1:** Model input parameters used to establish a base scenario for enzymatic cellulose hydrolysis. Kinetic values were obtained from the reported activities of *Trichoderma reesei *cellulases.

Parameter and units	Value	Parameter and units	Value
Cellulose rod dimensions (glucose residues)	30 × 30 × 150	EG adsorption strength	9,999
EG, CBHI and CBHII adsorption probabilities (%)	90	CBHI adsorption strength	9,999
EG concentration (mM)	800^a^	CBHII adsorption strength	9,999
CBHI concentration (mM)	2,000^a^	K_i _CBHI-cellobiose (mM)	1.6^d^
CBHII concentration (mM)	600^a^	K_i _CBHII-cellobiose (mM)	1.6^d^
BG concentration (mM)	20^a^	K_i _EG-cellobiose (mM)	11^d^
EG walk period (ms)	1^b^	K_m _BG-cellobiose (mM)	0.5^e,f^
CBHI walk period (ms)	1^b^	k_cat _BG-cellobiose (s^-1^)^h^	40^e^
CBHII walk period (ms)	1^b^	K_i _BG-glucose (mM)	0.5^f^
EG activity period^g^/s.d. (ms)	100/1	Timesteps	150,000
CBHI activity period/s.d. (ms)	500/1^c,d^	Iterations per sample	100
CBHII activity period/s.d. (ms)	500/1^c,d^	Maximum model life (s)	150
CBHI processivity/s.d. (reactions)	100/20 ^c^	Sample period (ms)	250
CBHII processivity/s.d. (reactions)	100/20 ^c^	EG, CBHI, CBHII and BG lifetime (s)	0 (infinite)

The following values were taken from or extrapolated from these literature sources: ^a ^[60]; ^b ^[34]; ^c ^[36]; ^d ^[58]; ^e ^[57]; ^f ^[59]; ^g ^activity period = 1/k_cat _for enzyme activities against solid substrates; ^h ^k_cat _values given for BG only, EG and CBH solution activities = 0 for the base case. BG: β-glucosidase; CBH: cellobiohydrolase; EG: endoglucanase; s.d.: standard deviation.

#### Varying the concentrations of EG and CBH

Elevated enzyme concentrations may result in an increased rate of cellulose hydrolysis but may also contribute to crowding on the cellulose surface, causing a reduction in hydrolysis; these potential outcomes were investigated using Cellulase 4D. The concentrations of all enzymes were varied by factors of 0.25, 0.5, 2, 3 and 4 in comparison to the concentrations used in the base case (= 1, Table 1). The production of saccharide species was monitored over time. In addition to this, the concentrations of EG alone were varied while CBH and BG were held at the base case concentrations to evaluate the effect that the amount of this enzyme had on glucose and cellobiose production.

#### Varying the adsorption strengths of EG and CBH

As described earlier, the adsorption strength parameter describes the probability of an enzyme spontaneously desorbing from the cellulose surface at any particular timestep. The selection of a parameter value of one causes the enzyme to desorb from cellulose in the next timestep, and at 10,000, the enzyme never desorbs unless it is on a section of cellulose that becomes soluble. For this scenario the absorption strength parameter was varied between 10,000 and 9,700 for EG and CBH enzymes and compared with the base case where the value was set at 9,999, and the production of saccharides over time was measured. The simulations were re-conducted varying the CBHI and EG adsorption strength parameters individually while maintaining the other enzymes at base case strengths.

#### Varying catalytic activities of EG and CBH

A complementary experiment to increasing the EG concentration while holding the others constant, as described above, was to increase the catalytic rate at which EG hydrolyzed cellulose. This was assessed by decreasing the time it took for each EG enzyme to complete a hydrolysis reaction after locating an appropriate reaction point. In this scenario there was potential for the accumulation of saccharide products that may result in increased competitive inhibition of hydrolysis. Further, the adsorption strength of EG was lowered to determine any effect this may have on crowding and saccharide production at various EG reaction periods. Similarly, the CBH reaction period was incrementally reduced from that of the base case, with all other parameters held at base case values, to determine any effect on the production of glucose and cellobiose over time.

### Statistical analysis

Concentration of glucose over time for replicate experiments from the base case and all scenarios were downloaded into Microsoft Excel spreadsheets. The final glucose concentrations for each scenario were examined for differences by one-way analysis of variance; where significant differences were found within a treatment, posthoc Student's t-tests with significance level of *P <*0.05 were conducted comparing glucose production between conditions.

## List of abbreviations and terms

BG: β-Glucosidase; CBHI: 1,4-β-D-cellobiohydrolase(exoglucanase) 1; CBHII: 1,4-β-D-cellobiohydrolase II; CBM: carbohydrate binding module; count_EG_: total number of EG in solution; EG: 1,4-β-D-endoglucanase; *k*_cat_: number of reactions per second of an enzyme working at v_max_; *k*_catSD_: Standard deviation of *k*_cat_: K_i_: dissociation constant - the concentration of inhibitor at which the activity of an enzyme is reduced to half (*v* = 1/2v_max_); *K*_m_: Michaelis constant - the concentration of substrate at which the activity of an enzyme is reduced to half (*v* = 1/2v_max_); v: reaction velocity for mean-field solution reactions (Equation 2); v_max_: maximum rate at which an enzyme catalyzes a reaction.

## Competing interests

The authors declare that they have no competing interests.

## Authors' contributions

ACW conceived the idea, designed the program, wrote the initial C++ code, conducted the scenarios and drafted the manuscript. BAL wrote the GUI, modified CUDA Particles for the 3D graphics display and assisted with some of the algorithm development. VSH contributed to experimental design of the program, ideas for scenarios and drafting the manuscript. All authors suggested modifications to the draft-read preliminary versions and final version, and approved the final manuscript.
